# Three Dimensional Quantitative Structure-Activity Relationship of 5*H*-Pyrido[4,3-b]indol-4-carboxamide JAK2 Inhibitors

**DOI:** 10.3390/ijms140612037

**Published:** 2013-06-05

**Authors:** Xiaoyun Wu, Shanhe Wan, Jiajie Zhang

**Affiliations:** School of Pharmaceutical Sciences, Southern Medical University, Guangzhou 510515, China; E-Mail: wansh@163.com

**Keywords:** 3D-QSAR, CoMFA, CoMSIA, JAK2 inhibitor, 5*H*-pyrido[4, 3-b]indol-4-carboxamide

## Abstract

Janus kinase 2 (JAK2) is an intracellular nonreceptor tyrosine kinase that belongs to the JAK family of kinases, which play an important role in survival, proliferation, and differentiation of a variety of cells. JAK2 inhibitors are potential drugs for the treatment of myeloproliferative neoplasms. The three dimensional quantitative structure-activity relationships have been studied on a series of JAK2 inhibitors by comparative molecular field analysis (CoMFA), and comparative molecular similarity indices analysis (CoMSIA). The CoMFA model had a cross-validated coefficient *q*^2^ of 0.633, and the relation non-cross-validated coefficient *r*^2^ of 0.976. The *F* value is 225.030. The contributions of steric and electrostatic fields to the activity are 55.2% and 44.8%, respectively. For the CoMSIA study, the *q*^2^, *r*^2^, and *F* values of the model are 0.614, 0.929, and 88.771, respectively. The contributions of steric, electrostatic, hydrophobic, hydrogen bond donor, and hydrogen bond donor fields to the activity are 27.3%, 23.9%, 16.4%, 21.7%, and 10.7%, respectively. The CoMFA and CoMSIA models showed strong predictive ability, and the 3D contour plots give the basis on the structure modification of JAK2 inhibitors.

## 1. Introduction

Janus kinase 2 (JAK2) is an intracellular nonreceptor tyrosine kinase that belongs to the JAK family kinases (JAK1, JAK2, JAK3, and TYK2), which is important in many of cellular signaling pathways [1–3]. JAK2 is essential for hematopoiesis, platelet formation, and other functions that are important in cellular survival, proliferation, and differentiation. In 2005, several groups independently reported the discovery of a somatic mutation of the gene encoding JAK2 in a high proportion of patients with myeloproliferative neoplasms (MPNs) : >95% for polycythemia vera (PV), and 50% for essential thrombocythemia (ET) and primary myelofibrosis (PMF) [4–8]. A single valine to phenylalanine mutation at position 617, located in the pseudokinase domain thought to negatively regulate the adjacent kinase domain, results in a constitutively active JAK2 tyrosine kinase. The Jak2^V617F^ mutation occurs in over 90% of PV patients and a large subset of ET and PMF patients. Therefore, JAK2 has been investigated in recent years as a potential therapeutic target for the treatment of MPNs. In fact, several classes of JAK2 small molecule inhibitors have been developed and are being tested in clinical trials for the treatment of MPNs [9–12]. Recently, a library of potent 5*H*-pyrido[4,3-b]indol-4-carboxamide JAK2 Inhibitors have been reported [13]. In order to design new compounds based on the structure of 5*H*-pyrido[4,3-b]indol-4-carboxamide, with excellent inhibitory activity for JAK2, quantitative structure-activity relationship (QSAR) techniques are useful methods. QSAR, which quantitatively correlates the variations in biological activity with the properties or molecular structures, is one of the most effective approaches for designing new chemical identities and understanding the action mechanisms of drugs [14,15]. In order to establish the QSAR model, suitable statistical methods are applied to establish correlations between chemical structures and their biological activities, such as Multiple Linear Regression (MLR), Principal Component Analysis (PCA), Partial Least Squares (PLS), artificial neural networks (ANN), Spectral-SAR Algorithm [16,17], and so on. When a model is established, the findings can be used to predict the properties of new compounds and see which structural factors influence those properties. In the present study, we performed molecular modeling studies on 5*H*-pyrido[4,3-b]indol-4-carboxamide JAK2 inhibitors using 3D-QSAR approaches, including comparative molecular field analysis (CoMFA), [18] and comparative molecular similarity indices analysis (CoMSIA) [19], to investigate the key structural features affecting the inhibitory activities.

## 2. Results and Discussion

### 2.1. Comparative Molecular Field Analysis (CoMFA) and Comparative Molecular Similarity Indices Analysis (CoMSIA) Results

**Figure 1:**
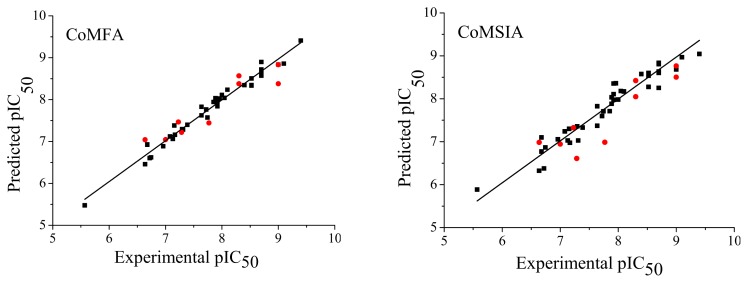
Graphs of the experimental *versus* predicted pIC_50_ values of the training (■) and test ( 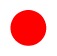) compounds from the CoMFA and CoMSIA models.

The 3D-QSAR studies were carried out using 5*H*-pyrido[4,3-b]indol-4-carboxamide derivatives, which are reported to be JAK2 inhibitors. The whole dataset was partitioned into a training set of 40 and a test set of nine compounds at random, with bias given to both chemical and biological diversity in both the training set and the test set molecules. The statistical results of the CoMFA and CoMSIA 3D-QSAR models are presented in Table 1. The CoMFA model gave a cross-validated correlation coefficient *q*^2^ of 0.633, an optimal number of principal components (N) of 6, and a non-cross-validated correlation coefficient *r*^2^ of 0.976. The corresponding contributions of steric and electrostatic fields were 55.2% and 44.8%, respectively. The CoMSIA model gave a cross-validated correlation coefficient *q*^2^ of 0.614, an optimal number of principal components of 5, and a non-cross-validated correlation coefficient *r*^2^ of 0.929. The corresponding contributions of steric, electrostatic, hydrophobic, hydrogen bond donor, and acceptor fields were 27.3%, 23.9%, 16.4%, 21.7%, and 10.7%, respectively. Both the CoMFA and CoMSIA models were satisfactory from the viewpoint of statistical significance. The activities of the 40 training compounds were predicted with the constructed CoMFA and CoMSIA models. The predicted pIC_50_ values are shown in Table 2 and Figure 1. It can be seen that the predicted pIC_50_ values were in good agreement with the experimental values, indicating that the obtained CoMFA and CoMSIA models had strong predictive ability.

### 2.2. Y-Randomization Test

The model was validated by applying the Y-randomization test. Several random shuffles of the Y vector were performed and the results are shown in Table 3. The low *q*^2^ and *r*^2^ values indicate that the good results in our original model are not due to a chance correlation or structural dependency of the training set.

### 2.3. Predictive Ability of Quantitative Structure-Activity Relationship (QSAR) Models

The predictive powers of the CoMFA and CoMSIA models were validated by the nine test compounds. The predicted pIC_50_ values were found to be in good agreement with the experimental data within an acceptable error range (Table 2 and Figure 1). The predictive correction coefficients of the CoMFA and CoMSIA models were 0.862 and 0.735, respectively. This result indicates that the CoMFA and CoMSIA models may be used to predict the inhibitory activities of novel 5*H*-pyrido[4,3-b]indol-4-carboxamide derivatives as JAK2 Inhibitors.

### 2.4. Contour Analysis

**Figure 2:**
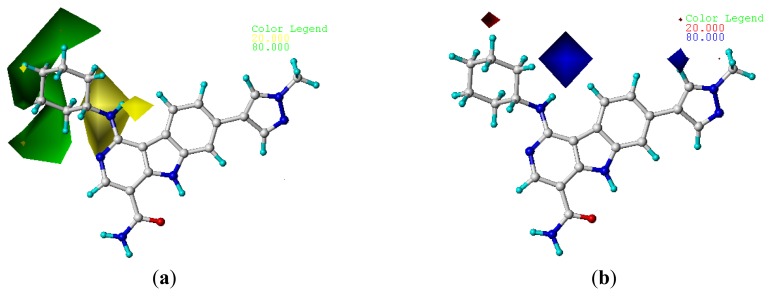
CoMFA StDev*Coeff contour maps. (**a**) Favorable (green) and unfavorable (yellow) steric fields; (**b**) Electropositive (blue) and electronegative (red) fields. Compound **22** was overlaid in each map. (**a**) (**b**)

**Figure 3:**
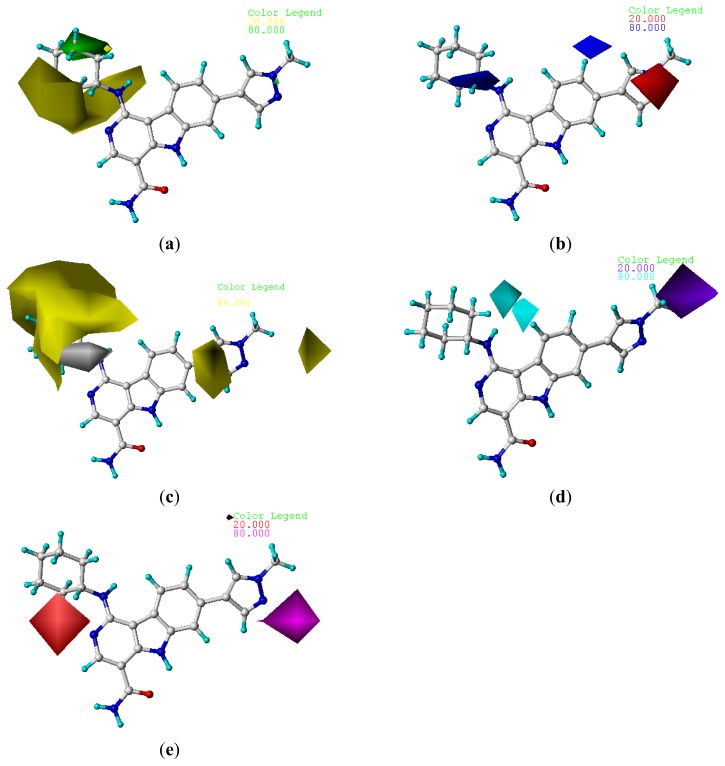
StDev*Coeff contour maps. (**a**) Favorable (green) and unfavorable (yellow) steric fields; (**b**) Electropositive (blue) and electronegative (red) fields; (**c**) Favorable (yellow) and unfavorable (gray) hydrophobic fields; (**d**) Favorable (cyan) and unfavorable (purple) hydrogen bond donor fields; (**e**) Favorable (magenta) and unfavorable (red) hydrogen bond acceptor fields. Compound **22** was overlaid in each plot. (**a**) (**b**) (**c**) (**d**) (**e**)

To visualize the results of the CoMFA and CoMSIA models, 3D coefficient contour maps were generated. The CoMFA and CoMSIA results were graphically interpreted by the field contribution maps using the StDev*Coeff field type. The contour maps of CoMFA (steric and electrostatic) and CoMSIA (steric, electrostatic, hydrophobic, hydrogen bond donor, and acceptor fields) are shown in Figures 2 and 3, respectively. Compound **22** was displayed in the map in aid of visualization. All the contours represented the default 80% and 20% level contributions for favorable and unfavorable regions, respectively.

#### 2.4.1. CoMFA Contour Maps

The CoMFA contour maps of the steric and electrostatic fields are shown in Figure 2. In the map of the steric field, the green contours represent the regions in which bulky groups confer an increase in the activity, whereas the yellow ones represent the regions where bulky groups may lead to a decrease in the activity. Similarly, in the map of electrostatic field, the blue contours indicate the regions where electropositive substitution increases the inhibitory activity, whereas the red contours indicate the regions where electronegative substitution increases the activity. In the CoMFA steric contour map (Figure 2a), a large green contour on the top of cyclohexane group of compound **22** suggests that the introducing of bulky groups at this position would increase the activity. Consistent with this, compounds bearing bulky groups at this position, for example, compounds **10**–**13**, showed high activities, whereas the ones bearing small groups at the same position, for example compounds **1** and **2**, showed low activities. A yellow contour near the cyclohexane group suggests that introducing of bulky groups at these positions would decrease the activity. For example, derivative **16** (IC_50_ = 2700 nM) was 14-fold less active than derivative **15** (IC_50_ = 190 nM). In addition, big yellow contours near the R_4_ substitute suggest that steric bulkiness is unfavorable by the model. This is in agreement with the fact that compound **19**, with chloro substituent, showed decreased activity. In the CoMFA electrostatic contour map, small blue contours near R_3_ or R_4_ of compound **22** indicates that introducing of electropositive groups around this position would increase the inhibitory activity. For example, compound **11** with electropositive hydrogen showed higher activity than the corresponding compounds **11** with electronegative chloro substituent.

#### 2.4.2. CoMSIA Contour Maps

The CoMSIA contour maps of the CoMSIA model are shown in Figure 3. The steric and electrostatic contour maps (Figure 3a,b) are quite similar to those of CoMFA model discussed above. Therefore, our following discussion will focus on the hydrophobic, hydrogen bond donor, and acceptor fields.

Figure 3c shows the hydrophobic contour maps in which yellow and gray contours indicate the regions where hydrophobic and hydrophilic groups are favored by the model, respectively. A large yellow contour around the cyclohexane group of compound **22** indicates that hydrophobic substituent at this position would increase the activity. This hydrophobic interaction may play a crucial role in improving of the binding affinity, since it is also observed that the cyclohexane occupied the hydrophobic pocket surrounded by the side chains Val863 and Leu983 [13]. In addition, two yellow contours around R_2_ substituent indicate that hydrophobic substituent at this position would increase the activity.

The CoMSIA hydrogen bond donor and acceptor contour plots are shown in Figure 3d,e, respectively. The cyan contours represent the regions where hydrogen bond-donating groups increase the activity, whereas the purple contours represent the regions where hydrogen bond-donating groups decrease the activity. Similarly, the magenta contours indicate the regions where hydrogen bond-accepting groups increase the inhibitory activity, whereas the red contours indicate the regions where hydrogen bond-accepting groups decrease the activity. The cyan contour near the R_1_ substituent indicates that hydrogen bond-donating groups are favored. This is well consistent with the observations that derivative **16** having a CH_2_CH_3_ to replace the hydrogen of compound **15** led to 14-fold decreases in the activities. A purple contour located on the methylpyrazole of R_2_ substituent suggests that hydrogen bond-donating groups are disfavored in this region. For example, compounds **24**, **41**, and **45** showed higher activity than the corresponding compounds **22**, **36**, and **37**, respectively. A magenta contour located on the R_2_ substitute suggests that hydrogen bond-accepting groups are favored in this region. This is evident from the fact that compound **24** was more active than compound **25**. A red contour located on the R_4_ substituent suggests that hydrogen bond-accepting groups are disfavored in this region. For example, compound **38** was less active than the optical isomer 3**7**, because the CF_3_ of **38** falls into the red contour.

### 2.5. Design of New Inhibitors

As shown above, the CoMFA and CoMSIA have provided detailed insight into the key structural requirements for potent activities of the inhibitors of this class. To demonstrate the practical values of these structure-activity relationships, a series of new inhibitors were designed, and their pIC_50_ values were predicted with the established CoMFA and CoMSIA models (Table 4). The designed molecules exhibited good predicted pIC_50_ values in CoMFA or CoMSIA models. This result strongly suggested that our models can be used to guide the design of new inhibitors of this class. The detailed synthesis and evaluation of the representative molecule are shown in supplementary information.

## 3. Experimental Section

### 3.1. General

The crystallographic coordinates of JAK2 in complex with small-molecule inhibitor were obtained from the Brookheaven Protein Databank as entries 3RVG [13]. All the molecular modeling and calculations were performed using the Sybyl version 7.3 molecular modeling package (Tripos International, St. Louis, MO, USA) [20].

### 3.2. Data Set

Compounds **1**–**49** selected for the present study were taken from the literature [13], and served as the database in the molecular modeling. Their structures and inhibitory activities are listed in Tables 2 and 5. Among them, the 9 compounds that are asterisk labeled served as the test set, and the rest as the training set. The IC_50_ values (M) were converted to the corresponding pIC_50_ (=−logIC_50_) and used as dependent variables in the CoMFA and CoMSIA analyses.

### 3.3. Molecular Modeling

In the 3D-QSAR study, the selection of active conformations is a key step for CoMFA and CoMSIA studies. The X-ray crystallographic structure of small ligand **22** complexed with the JAK2 was selected as the bioactive conformation, which was used as the template to construct the 3D structures of the rest of the compounds in the data set. Structural energy minimization process was performed using the Tripos force field with a distance-dependent dielectric and Powell gradient algorithm with a convergence criterion of 0.001 kcal/mol. Partial atomic charges were calculated using the Gasteiger-Hückel method.

### 3.4. Molecular Alignment

**Figure 4:**
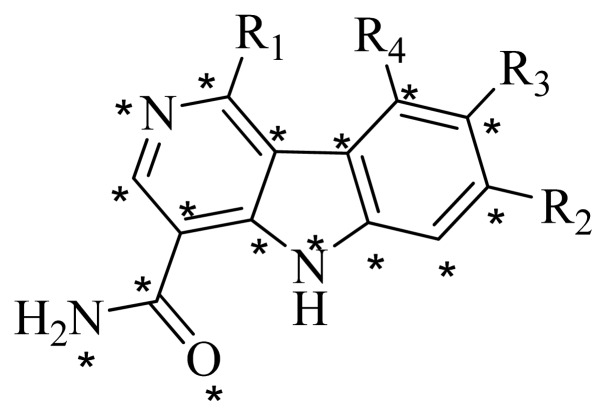
Structure of 5*H*-pyrido[4,3-b]indol-4-carboxamide derivatives, the asterisk indicate the atoms selected as the common substructure.

**Figure 5:**
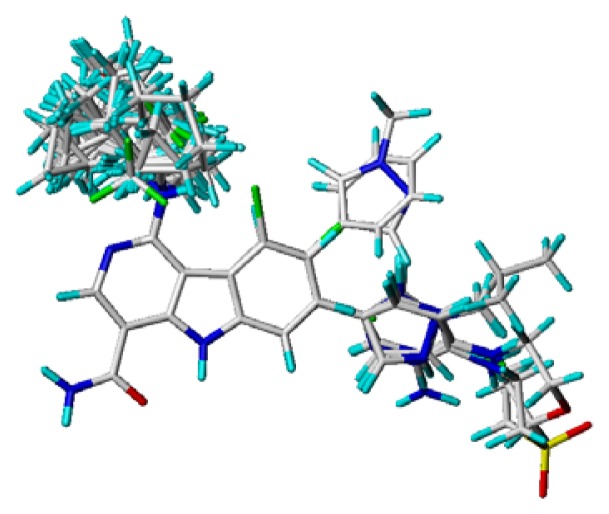
Superimposition of compounds for CoMFA and CoMSIA studies.

In the 3D-QSAR study, the alignment rule is also a key step. The predictive accuracy of the CoMFA and CoMSIA models and the reliability of the contour maps are directly dependent on the structural alignment rule. In the present study, the co-crystallized molecule **22** was chosen as a template to fit the remaining compounds in the training and test sets. Thus, all compounds in the data set were aligned to template molecule using the “align database” command in Sybyl, with the 5*H*-pyrido[4,3-b]indol-4-carboxamide as the common substructure (Figure 4). The aligned compounds are shown in Figure 5.

### 3.5. Generation of CoMFA and CoMSIA Models

Standard CoMFA and CoMSIA procedures were performed. A 3D cubic lattice was created automatically by extending at least 4 Å beyond all the investigated molecules in all the three axes (X, Y, and Z directions) with 2.0 Å grid spacing. The CoMFA steric (Lennard-Jones potential) and electrostatic (Coulomb potential) fields at each lattice were calculated using the standard Tripos force field method. A distance dependent dielectric constant of 1.0 was used, and an sp^3^ hybridized carbon atom with one positive charge and a radius of 1.52 Å served as a probe atom to calculate the steric and electrostatic fields. The default cutoff value of 30.0 kcal/mol was adopted.

Compared with CoMFA, CoMSIA methodology has the advantage of exploring the impacts of more fields. The CoMSIA method defines hydrophobic (H), hydrogen bond donor (D), and hydrogen bond acceptor (A) descriptors, in addition to the steric (S) and electrostatic (E) fields used in CoMFA. The CoMSIA fields were derived, according to Klebe *et al.* [19], from the same lattice box that was used in the CoMFA calculations, with a grid spacing of 2 Å, and a probe carbon atom with one positive charge and a radius of 1.0 Å as implemented in Sybyl. Arbitrary definition of cutoff limits were not required in CoMSIA method, wherein the abrupt changes of potential energy near the molecular surface were taken into account in the distance dependent Gaussian type functional form. The default value of 0.3 was used as the attenuation factor.

### 3.6. Partial Least Squares (PLS) Regression Analysis and Validation of QSAR Models

Partial least squares (PLS) approach was used to derive the 3D QSAR models. The CoMFA and CoMSIA descriptors were used as independent variables and the pIC_50_ values were used as dependent variables. CoMFA and CoMSIA column filtering was set to 2.0 kcal/mol to improve the signal-to-noise ratio. The leave-one-out (LOO) cross-validation was carried out to obtain the optimal number of components (N) and the correlation coefficient *q*^2^. The obtained N was then used to derive the final QSAR model and to obtain the non-cross-validation correlation coefficient *r*^2^, standard error of estimate (SEE), and *F*-value.

### 3.7. Y-Randomization Test of QSAR Models

The model was further validated by applying the Y-randomization test. “Y-randomization” is also known as the “Y-scrambling test”. This technique ensures the robustness of a QSAR model [21]. The dependent variable vector (pIC_50_) is randomly shuffled and a new QSAR model is developed using the original independent variable matrix. The new QSAR models (after several repetitions) are expected to have lower *r*^2^ and *q*^2^ values than the true value of original models. This method is usually performed to eliminate the possibility of chance correlation. If higher values are obtained, an acceptable 3D-QSAR model cannot be generated for a particular data set because of chance correlation and structural redundancy.

### 3.8. Predictive Correlation Coefficient of QSAR Models

To assess the predictive power of the derived 3D-models, a set of test compounds that had known biological activities was used to validate the obtained models. The predictive correlation *r*^2^_pred._ value was calculated using:

(1) $${r^{2}}_{\text{pred}.}=(\text{SD}-\text{PRESS})/\text{SD}$$

wherein SD is the sum of the squared deviations between the biological activities of the test compounds and the mean activities of the training compounds, and PRESS is the sum of the squared deviations between the experimental and the predicted activities of the test compounds.

## 4. Conclusions

In this study, 3D-QSAR analyses, CoMFA and CoMSIA, have been applied to a set of recently synthesized 5*H*-pyrido[4,3-b]indol-4-carboxamide derivatives as JAK2 Inhibitors. The CoMFA and CoMSIA models showed statistically significant results in terms of cross-validated coefficients and conventional coefficients. Their predictive capabilities were verified by the test compounds. The derived CoMFA and CoMSIA models showed predictive cross-validated coefficients of 0.976 and 0.929, respectively, and the activities of the training and test compounds were predicted with good accuracy. Based on the obtained structure-activity relationships, a series of new inhibitors were designed to have excellent activities, which were predicted with the developed CoMFA and CoMSIA models. Thus, these models may be expected to serve as a tool to guide the future rational design of 5*H*-pyrido[4,3-b]indol-4-carboxamide-based novel JAK2 Inhibitors with potent activities.

## Supplementary Information



## Figures and Tables

**Table 1:** The statistical results of comparative molecular similarity indices analysis (CoMSIA) and comparative molecular field analysis (CoMFA) models.

Model	*N*	*q*^2^	*r*^2^	SEE	F	*r*^2^_pred._	Field contribution				

							S	E	H	D	A
CoMFA	6	0.633	0.976	0.138	225.030	0.862	0.552	0.448	-	-	-
CoMSIA	5	0.614	0.929	0.234	88.771	0.735	0.273	0.239	0.164	0.217	0.107

*N*: the number of compounds used in the correlation; *q*^2^: Cross-validated correlation coefficient; *r*^2^: non-cross-validated correlation coefficient; *r*^2^_pred._: predictive correlation coefficient *r*^2^; SEE: standard error of estimate; F: the Fischer ratio; S: steric field; E: electrostatic field; H: hydrophobic field. D: hydrogen bond donor field; A: hydrogen bond acceptor field.

**Table 2:** The experimental and predicted activities (pIC_50_ in M) of the CoMFA and CoMSIA models.

No.	Experimental pIC_50_	Predicted pIC_50_(CoMFA)		Predicted pIC_50_(CoMSIA)	
	
		Pred.	Resid.	Pred.	Resid.
1	6.678	6.920	−0.243	7.098	−0.420
2 *	6.638	7.043	−0.405	6.982	−0.344
3	7.168	7.159	0.009	6.974	0.194
4	7.310	7.283	0.027	7.028	0.282
5 *	7.000	7.045	−0.045	6.944	0.056
6	7.131	7.061	0.070	7.029	0.102
7 *	7.284	7.219	0.065	6.609	0.675
8	6.678	6.930	−0.252	6.769	−0.091
9	7.081	7.122	−0.042	7.238	−0.157
10	7.292	7.295	−0.002	7.358	−0.066
11 *	7.770	7.443	0.326	6.986	0.784
12	7.721	7.763	−0.042	7.599	0.122
13	7.745	7.573	0.172	7.707	0.038
14	6.638	6.458	0.180	6.324	0.314
15	6.721	6.603	0.118	6.377	0.344
16	5.569	5.475	0.094	5.884	−0.315
17 *	7.229	7.466	−0.237	7.326	−0.097
18	7.638	7.625	0.014	7.371	0.268
19	6.959	6.887	0.072	7.057	−0.098
20	7.155	7.383	−0.228	7.300	−0.146
21	6.745	6.621	0.123	6.866	−0.122
22 *	8.301	8.568	−0.267	8.422	−0.121
23	7.886	8.034	−0.148	8.034	−0.148
24	7.886	7.996	−0.110	7.883	0.003
25	7.638	7.828	−0.189	7.827	−0.189
26 *	8.301	8.380	−0.079	8.048	0.253
27	7.387	7.398	−0.011	7.330	0.058
28	7.921	7.840	0.081	7.976	−0.055
29	7.921	7.935	−0.014	8.107	−0.186
30	7.854	7.943	−0.090	7.710	0.144
31	8.000	8.112	−0.112	7.980	0.020
32	9.000	8.838	0.162	8.683	0.317
33	7.959	8.025	−0.066	8.360	−0.402
34	8.699	8.675	0.024	8.254	0.445
35	8.523	8.506	0.016	8.600	−0.077
36	8.699	8.898	−0.199	8.807	−0.108
37	9.398	9.410	−0.012	9.042	0.356
38	8.046	8.042	0.004	8.180	−0.134
39	8.523	8.333	0.190	8.537	−0.015
40	7.921	7.996	−0.075	8.354	−0.433
41	8.523	8.348	0.175	8.275	0.248
42	8.699	8.654	0.045	8.835	−0.136
43	8.398	8.345	0.053	8.576	−0.178
44 *	9.000	8.378	0.622	8.764	0.236
45 *	9.000	8.835	0.165	8.504	0.496
46	8.699	8.718	−0.020	8.600	0.099
47	8.097	8.235	−0.138	8.169	−0.072
48	8.699	8.574	0.125	8.634	0.065
49	9.097	8.859	0.238	8.966	0.131

* Test set.

**Table 3:** *q*^2^ and *r*^2^ values after several Y-randomization tests.

Iteration	CoMFA		CoMSIA	
	
	*q*^2^	*r*^2^	*q*^2^	*r*^2^
1	0.465	0.821	0.419	0.511
2	0.458	0.546	0.393	0.506
3	0.488	0.678	0.501	0.577
4	0.325	0.435	0.379	0.495
5	0.302	0.420	0.331	0.453
6	0.216	0.347	0.247	0.386
7	0.269	0.589	0.313	0.422
8	0.313	0.430	0.343	0.464
9	0.281	0.389	0.303	0.417
10	0.338	0.435	0.295	0.412

**Table 4:** Structures and predicted pIC_50_ values of newly designed derivatives.

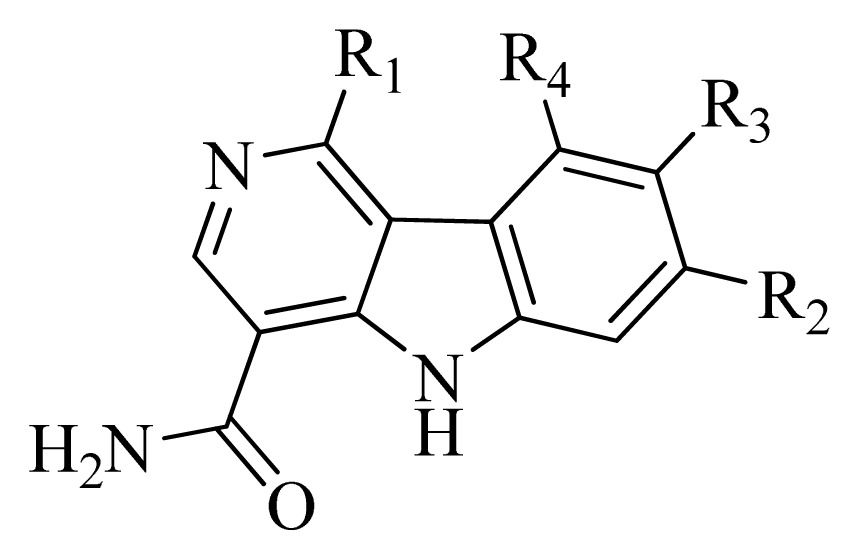						

No.	R_1_	R_2_	R_3_	R_4_	Predicted pIC_50_	

					CoMFA	CoMSIA
**D1**	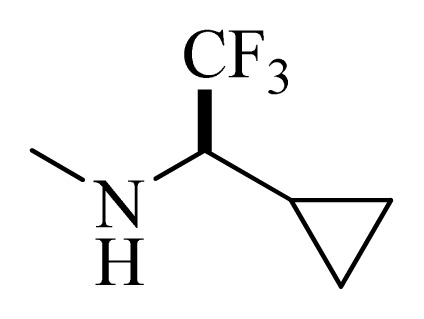		F	H	9.340	8.750
**D2**	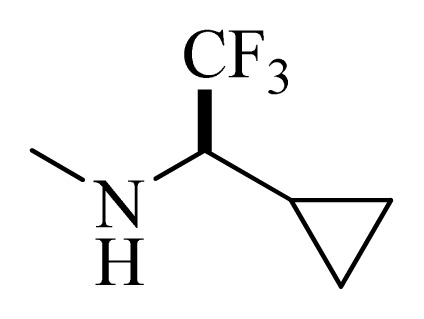		H	F	9.485	8.852
**D3**	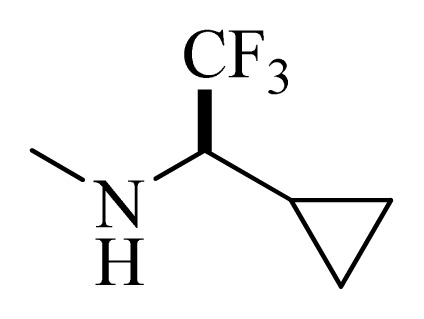		F	F	9.414	8.561
**D4**	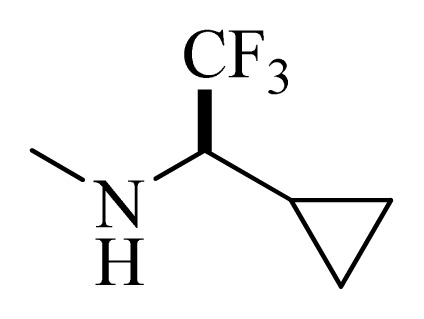		Me	H	8.978	9.380
**D5**	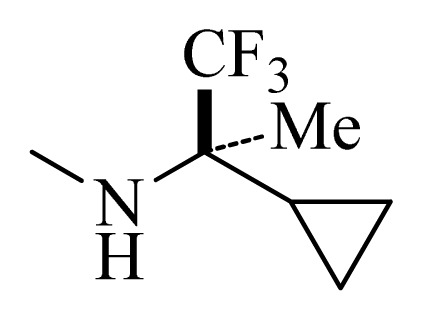		H	H	8.965	9.416
**D6**	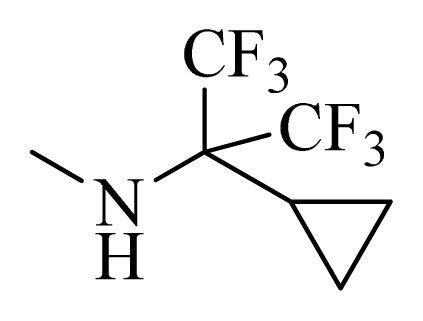		H	H	8.972	9.272
**D7**	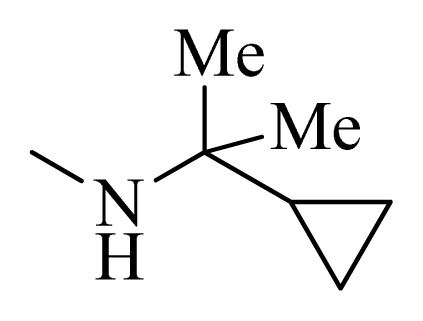		H	H	8.622	8.796
**D8**	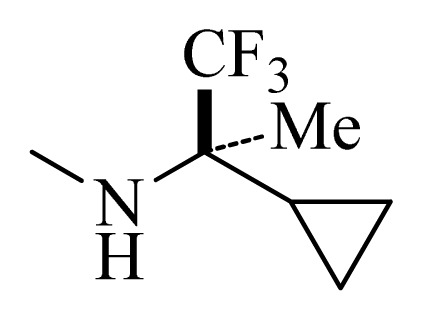		F	H	9.346	8.676
**D9**	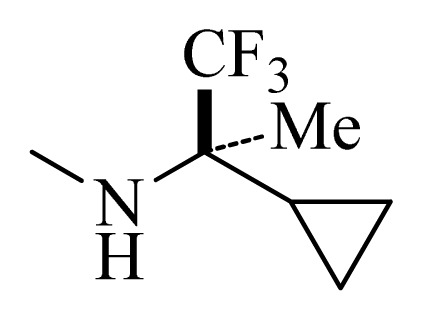		Me	H	9.169	8.617
**D10**	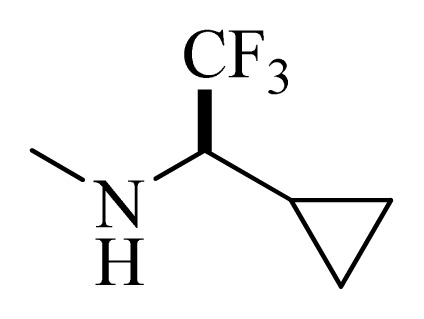	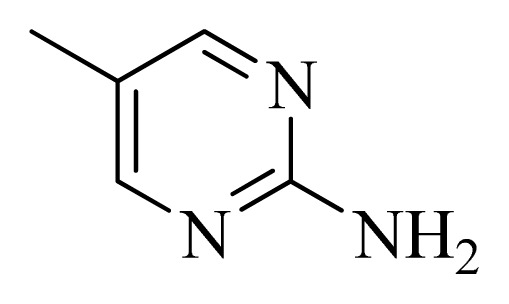	Me	H	8.905	8.816
**D11**	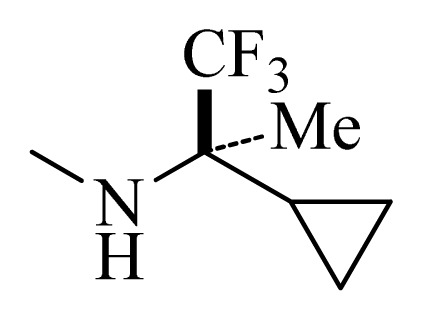	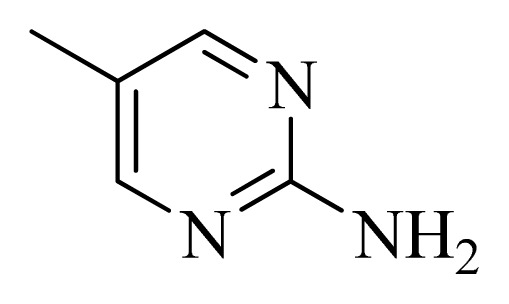	H	H	8.865	8.890
**D12**	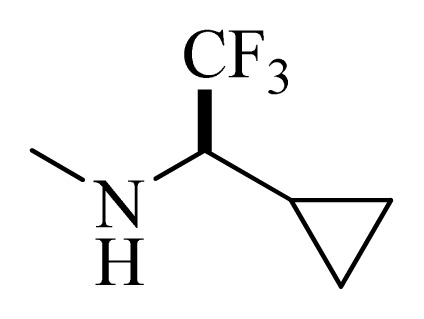	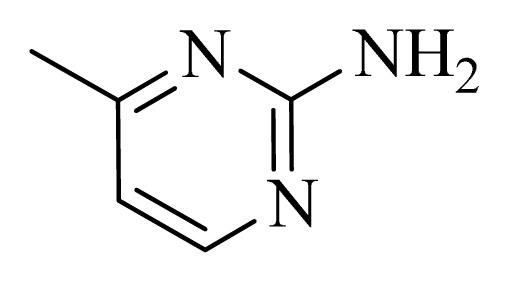	H	H	8.585	9.092
**D13**	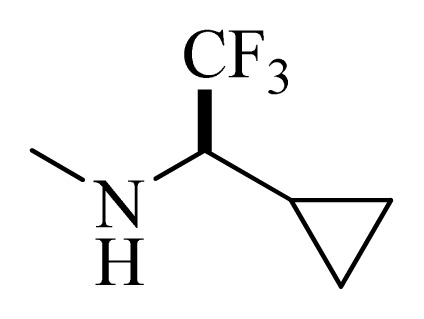	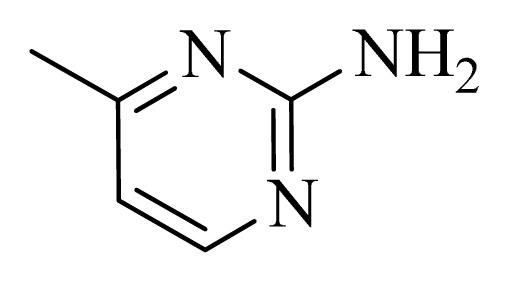	Me	H	8.547	9.032
**D14**	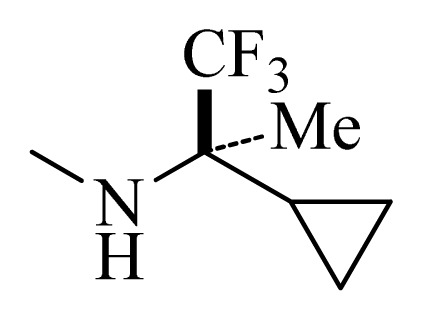	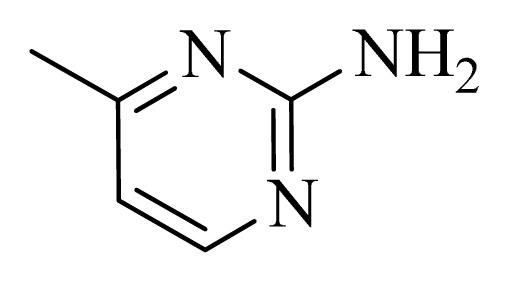	Me	H	8.554	8.956
**D15**	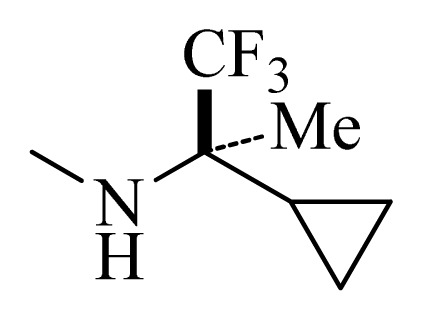	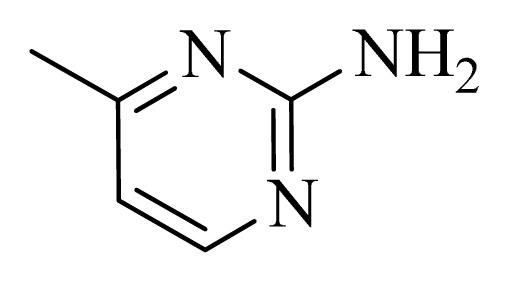	H	H	8.509	8.711

**Table 5:** The molecules of 5*H*-pyrido[4,3-b]indol-4-carboxamide derivatives.

No.	R_1_	R_2_	R_3_	R_4_
**1**	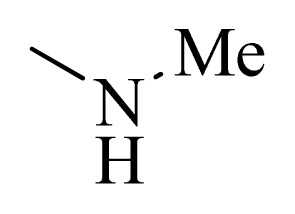	H	F	H
**2** *	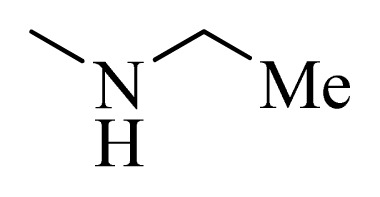	H	F	H
**3**	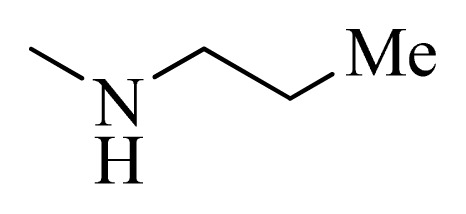	H	F	H
**4**	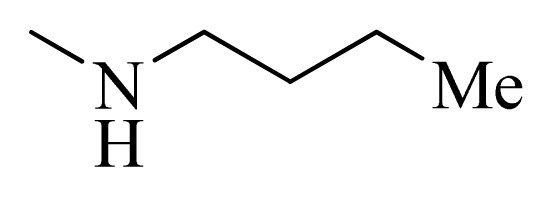	H	F	H
**5** *	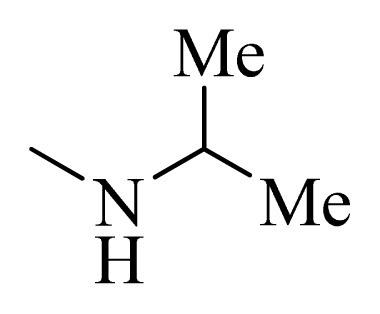	H	F	H
**6**	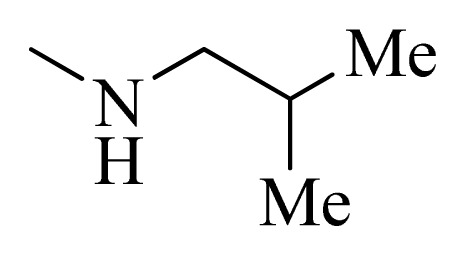	H	F	H
**7** *	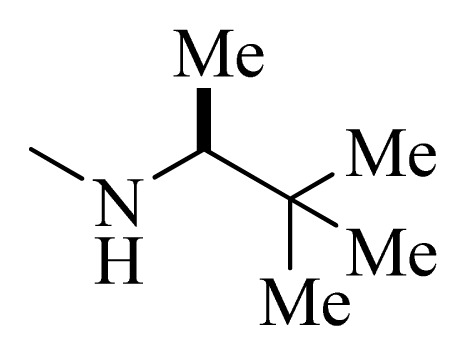	H	F	H
**8**	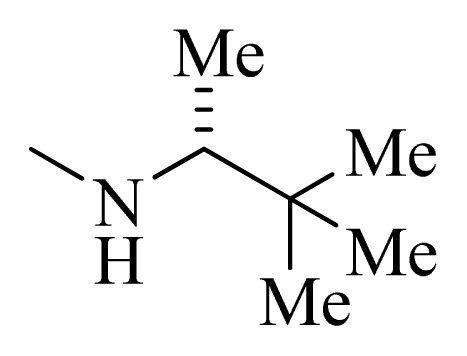	H	F	H
**9**	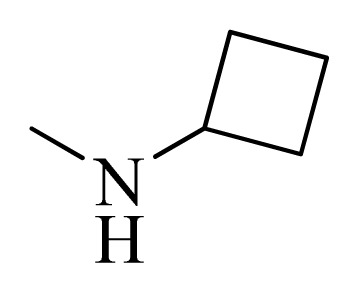	H	F	H
**10**	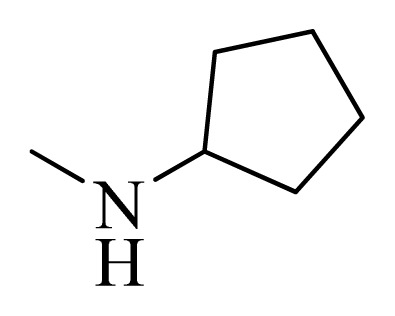	H	F	H
**11** *	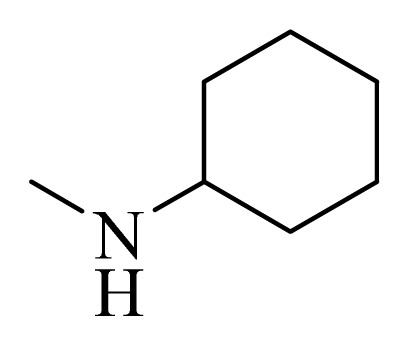	H	F	H
**12**	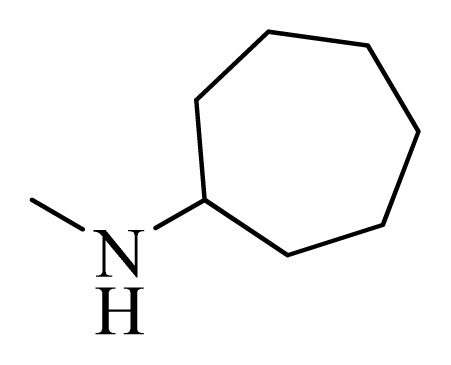	H	F	H
**13**	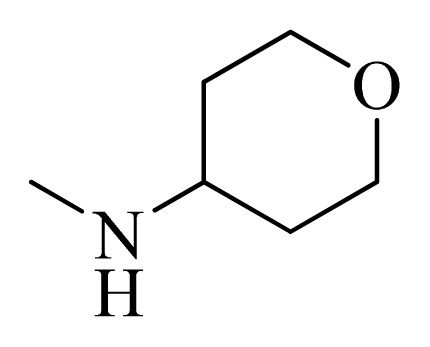	H	F	H
**14**	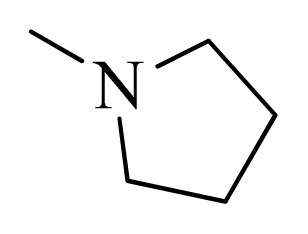	H	F	H
**15**	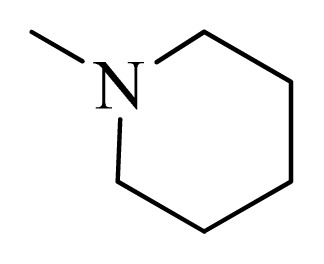	H	F	H
**16**	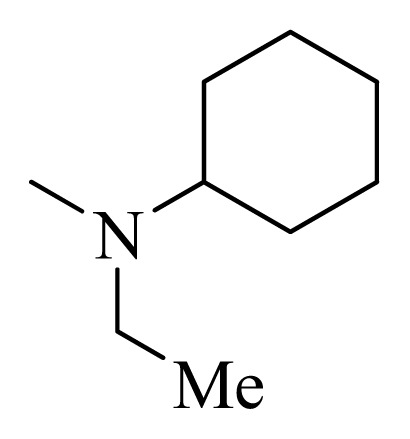	H	F	H
**17** *	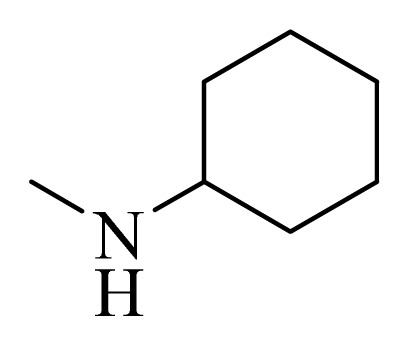	H	H	H
**18**	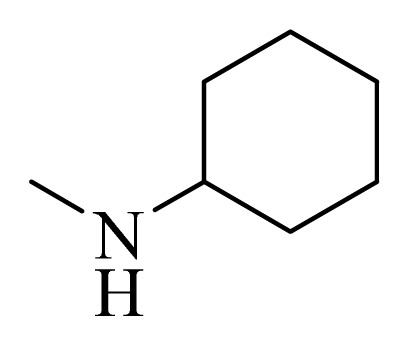	Cl	H	H
**19**	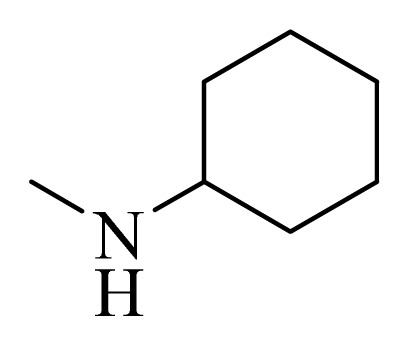	H	H	Cl
**20**	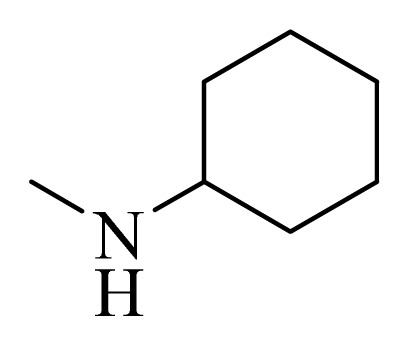	Phenyl	H	H
**21**	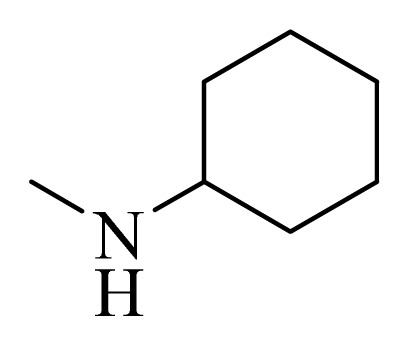	H	Phenyl	H
**22** *	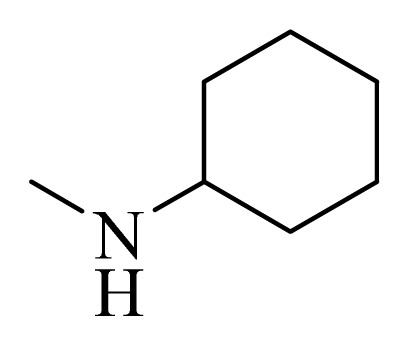		H	H
**23**	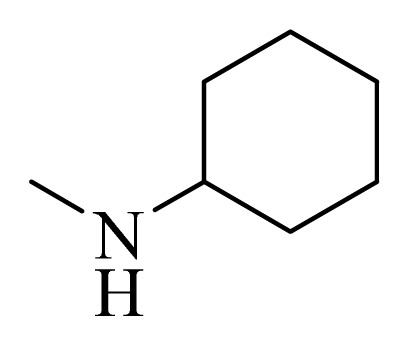	H		H
**24**	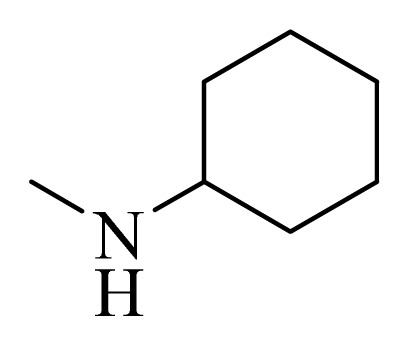	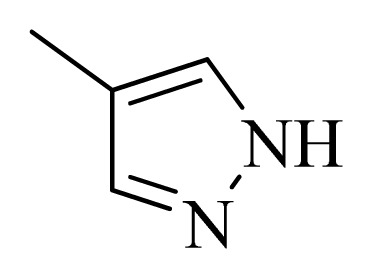	H	H
**25**	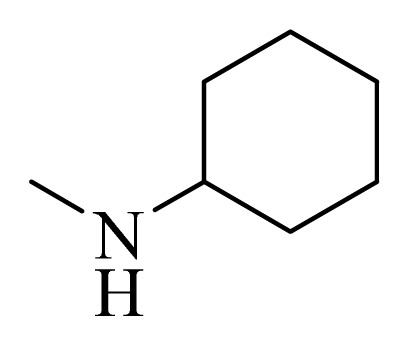	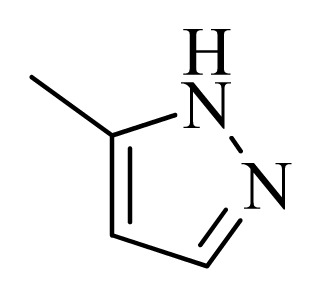	H	H
**26** *	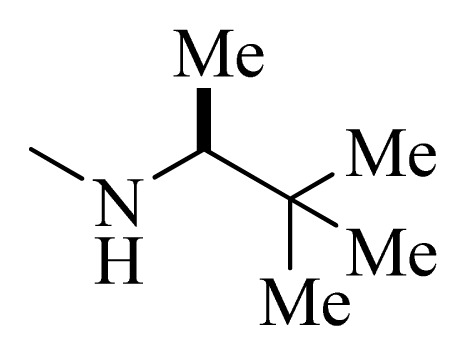		H	H
**27**	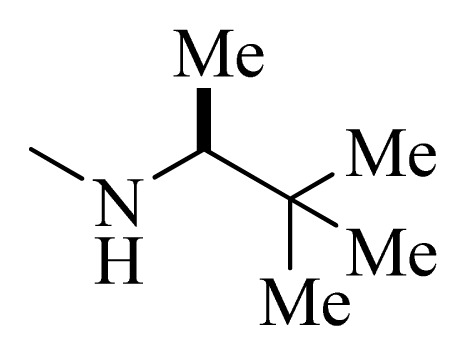	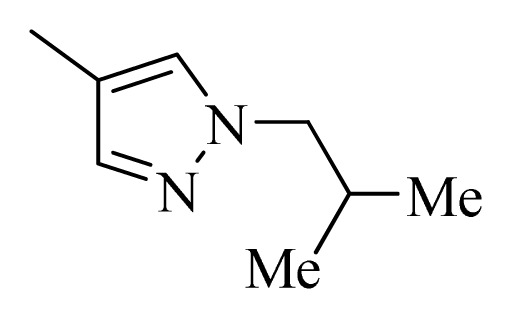	H	H
**28**	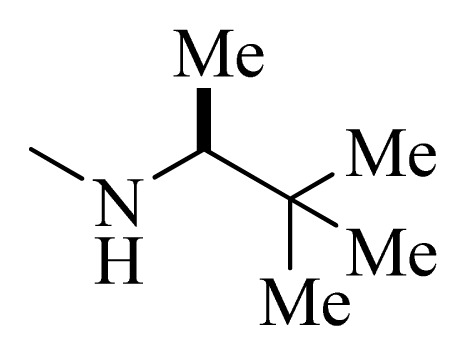	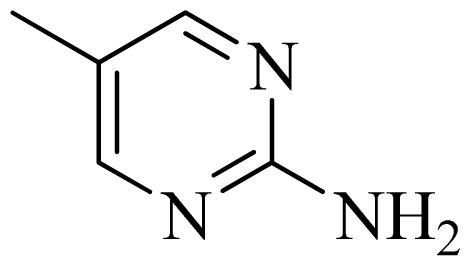	H	H
**29**	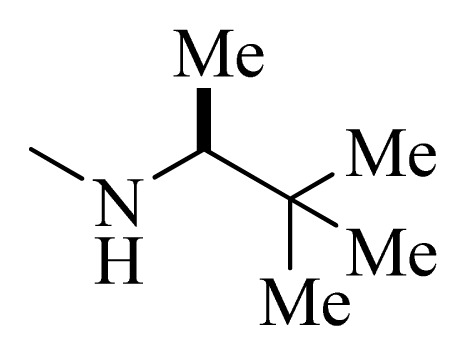	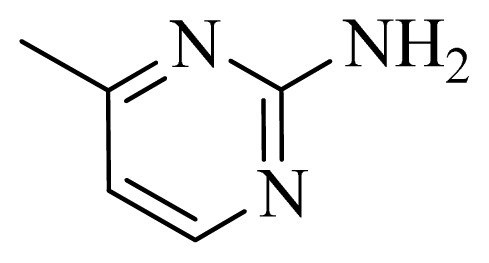	H	H
**30**	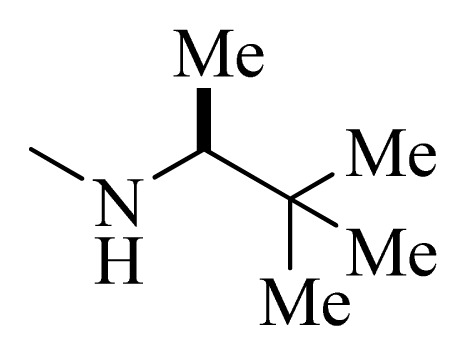	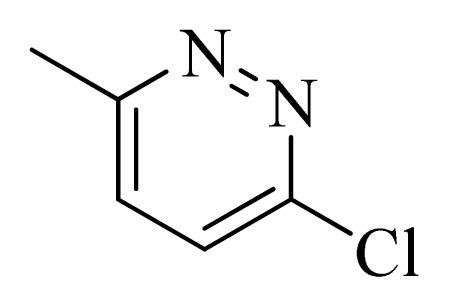	H	H
**31**	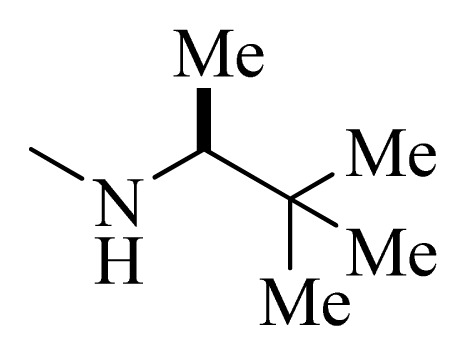	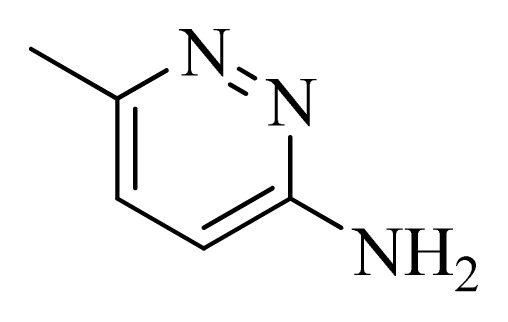	H	H
**32**	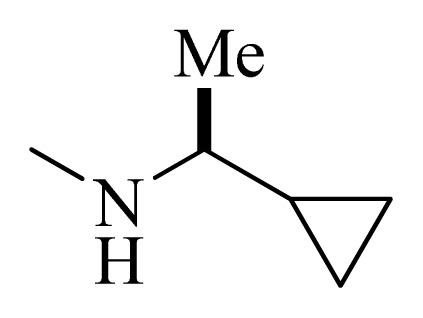		H	H
**33**	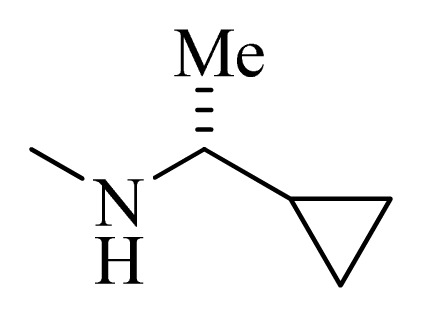		H	H
**34**	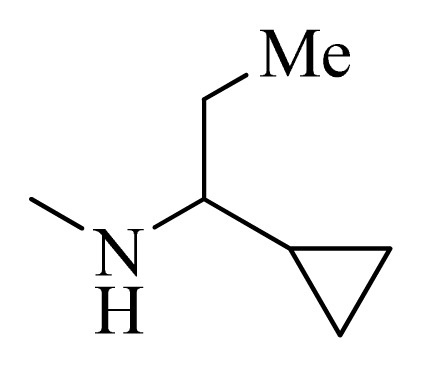		H	H
**35**	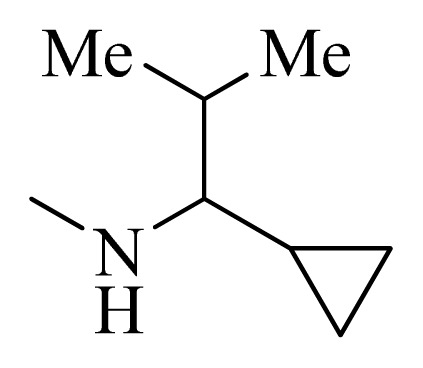		H	H
**36**	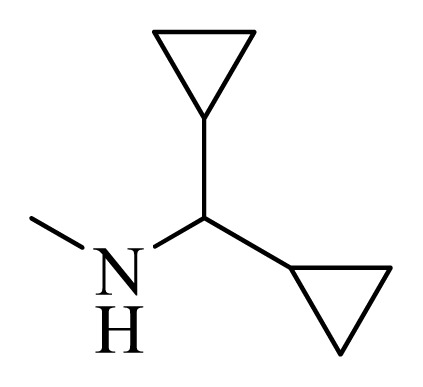		H	H
**37**	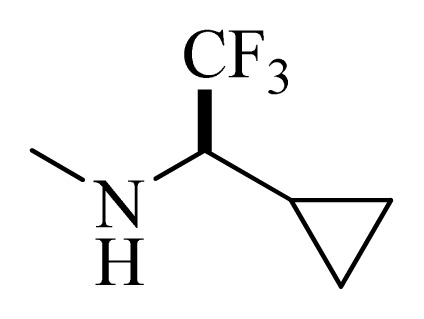		H	H
**38**	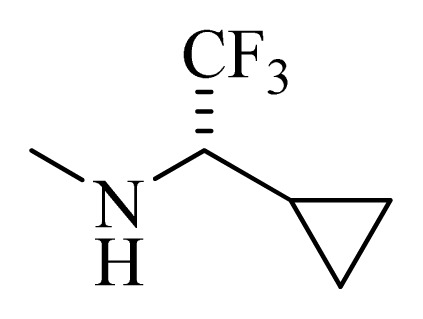		H	H
**39**	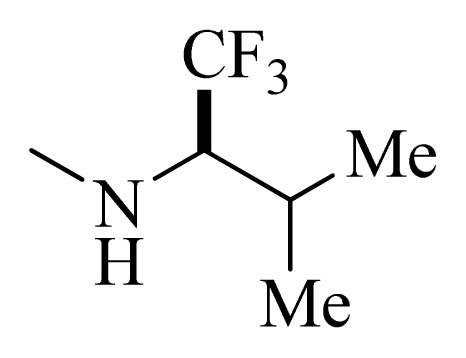		H	H
**40**	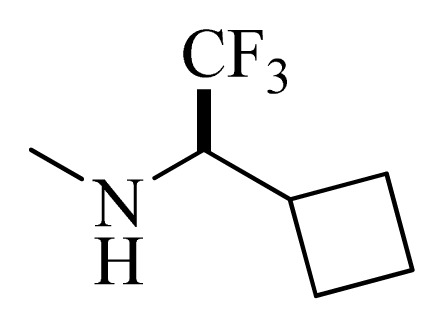		H	H
**41**	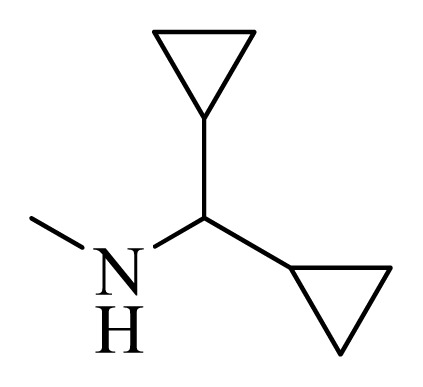	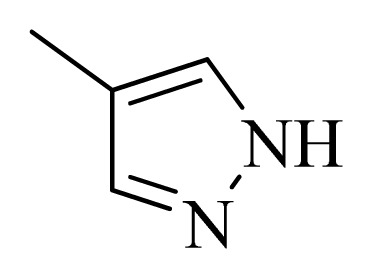	H	H
**42**	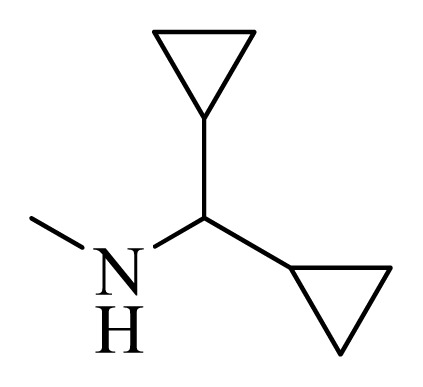	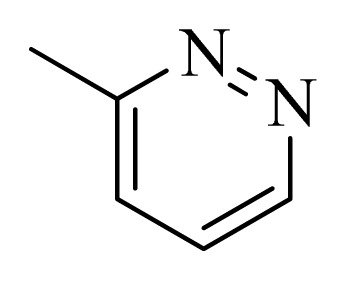	H	H
**43**	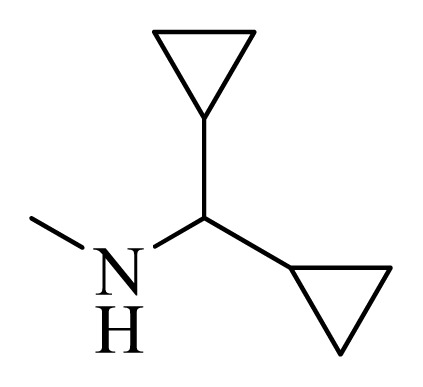	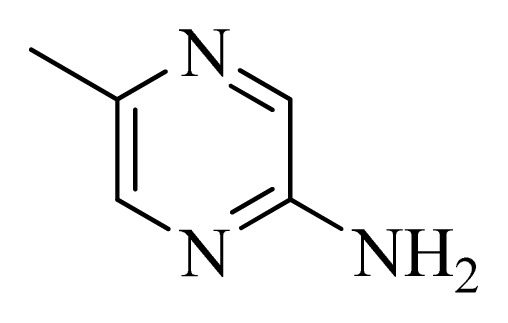	H	H
**44** *	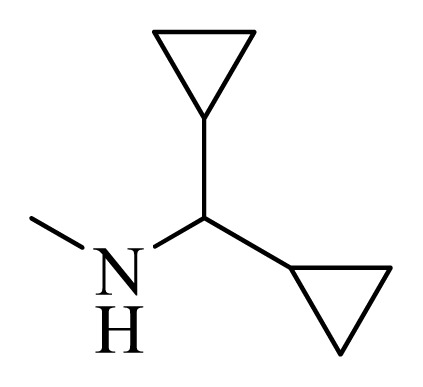	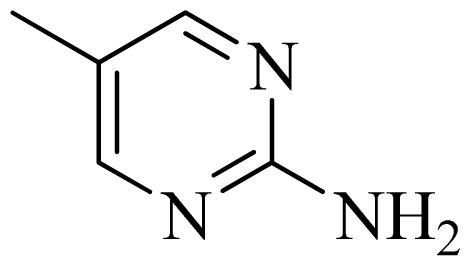	H	H
**45** *	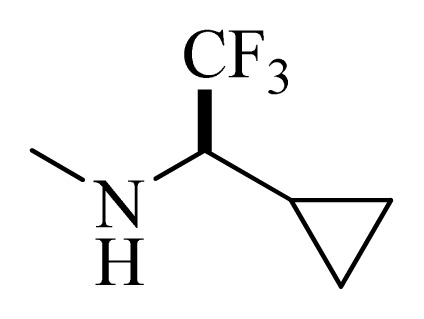	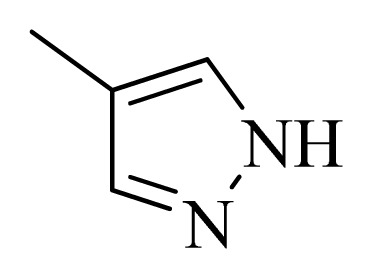	H	H
**46**	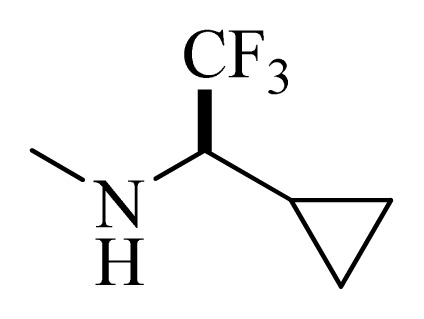	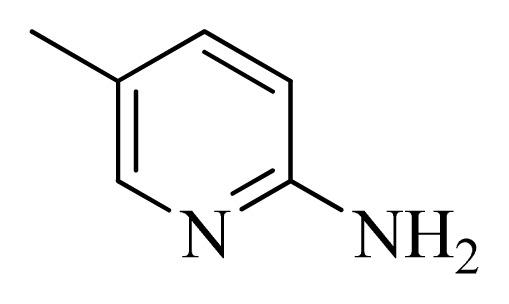	H	H
**47**	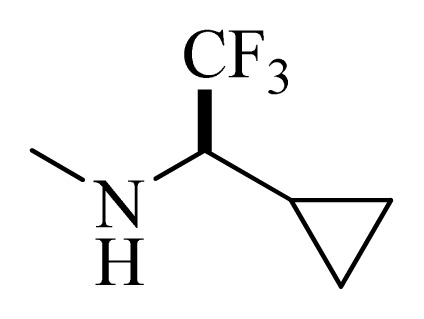	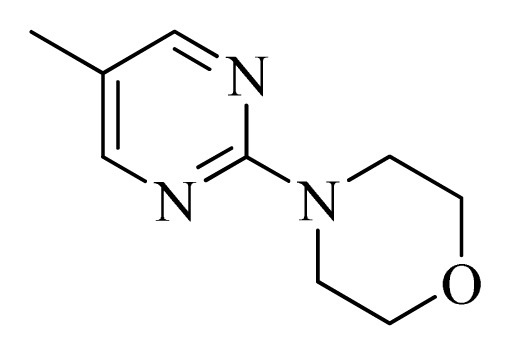	H	H
**48**	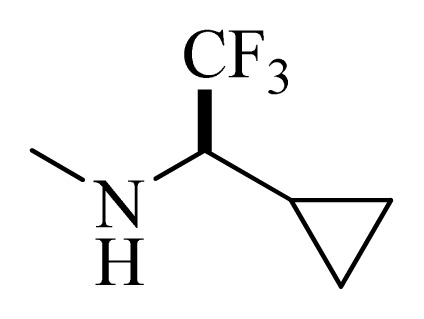	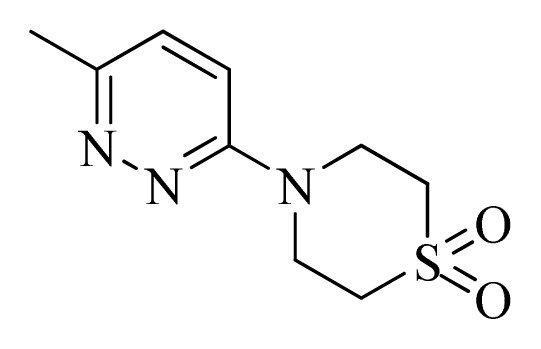	H	H
**49**	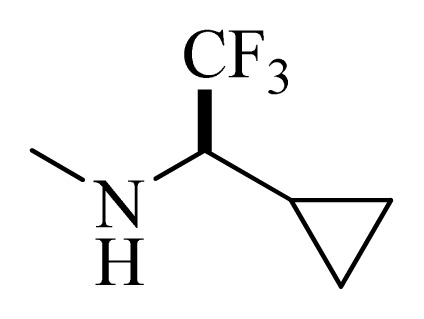	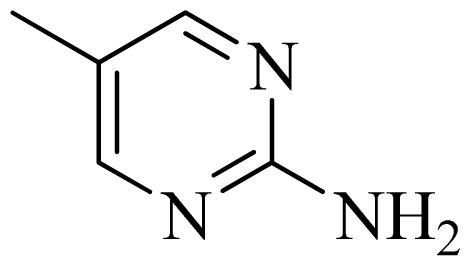	H	H

* Test set.
