# Supplementary material for: Three Dimensional Quantitative Structure-Activity Relationship of 5H-Pyrido[4,3-b]indol-4-carboxamide JAK2 Inhibitors
Source: Int J Mol Sci. 2013 Jun 5;14(6):12037–53. doi: 10.3390/ijms140612037 (PMC3709772; doi:10.3390/ijms140612037)
Supplement: Supplementary file 1 [file ijms-14-12037-s001.pdf]

Supplementary Information

Scheme 1. The synthesis of representative compound D2. Compound D2 was synthesized from Fischer indole which was synthesized beteeen 2,4-dihydropyridine and 3,5-difluorophenylhydrazine hydrochloride.

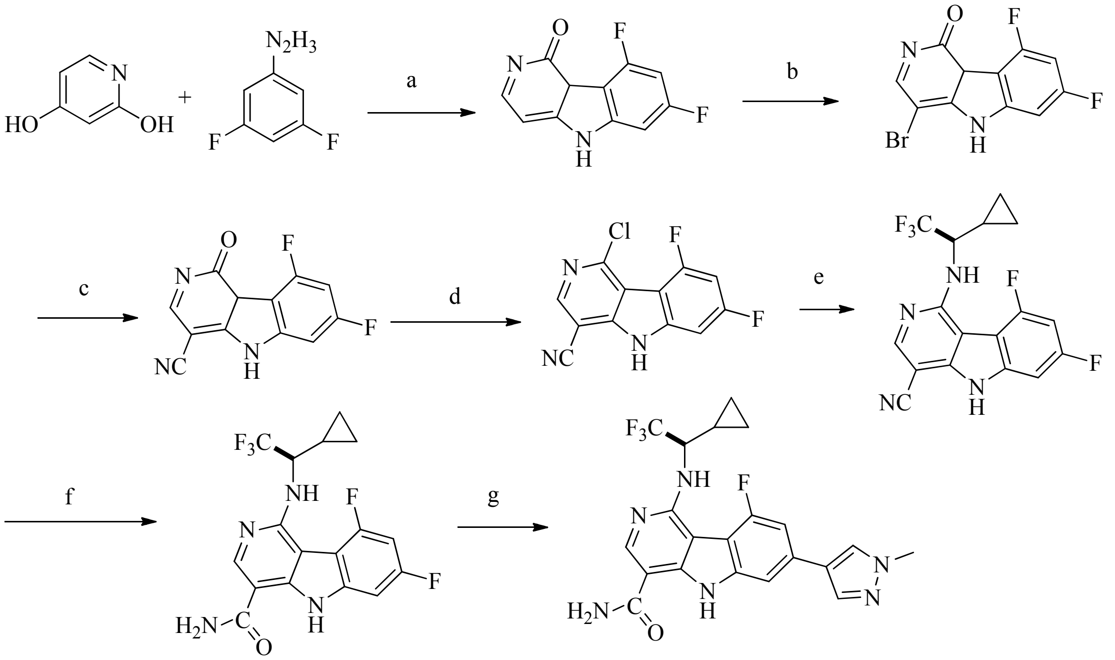

(**a**) Dean-Stark trap, Ph_2O; (**b**) NBS, DMF; (**c**) $Zn(CN)_2$, $Pd(PPh_3)_4$, DMF; (**d**) $POCl_3$; (**e**) (1R)-1-cyclopropyl-2,2,2-trifluoroethanaminium chloride, $Pd_2(dba)_3$, BINAP, NaO^tBu, DME; (**f**) Aqueous H_2O_2, K_2CO_3, DMSO; (**g**) 1-methyl-4-(4,4,5,5-tetra-methyl-1,3,2-dioxaborolan-2-yl)-1H-pyrazole , $Pd_2(dba)_3$, PCy_3, K_3PO_4.

MS(ESI) 447.4, ^{1}H-NMR (400MHz, DMSO-d6) δ 0.27–0.34 (m, 1H), 0.47–0.53 (m, 1H), 0.58–0.62 (m, 1H), 0.68–0.75 (m, 1H), 1.73–1.79 (m, 1H), 3.95 (s, 3H), 4.80–4.88 (m,1H), 6.90 (d, J = 8.8 Hz, 1H), 7.25 (br, s, 1H), 7.44 (s, 1H), 7.80 (s, 1H), 7.85 (s, 1H), 7.89 (br, s,1H), 8.09 (s, 1H), 8.44 (s, 1H), 11.50 (s, 1H).

IC_{50} values for inhibitors of JAK2 were determined using HTRF detection technology. 20 pM JAK2 was incubated in 50 mM Tris, 5 mM $MgCl_2$, 100 mM NaCl, 0.1 mg/mL BSA, 2 mM DTT, 5 μM ATP and 2 μM amino hexanoyl biotin-EQEDEPGDYFEWLE-NH2 for 60 min at ambient temperature. Inhibitors were tested at a final concentration of 5% DMSO.

© 2013 by the authors; licensee MDPI, Basel, Switzerland. This article is an open access article distributed under the terms and conditions of the Creative Commons Attribution license (http://creativecommons.org/licenses/by/3.0/).
